# Packed-Fiber Solid Phase-Extraction Coupled with HPLC-MS/MS for Rapid Determination of Lipid Oxidative Damage Biomarker 8-Iso-Prostaglandin F_2α_ in Urine

**DOI:** 10.3390/molecules27144417

**Published:** 2022-07-10

**Authors:** Ying Sun, Yan Yan, Xuejun Kang

**Affiliations:** 1 Key Laboratory of Child Development and Learning Science (Ministry of Education), School of Biological Science & Medical Engineering, Southeast University, Nanjing 210096, China; 230189182@seu.edu.cn; 2 Key Laboratory of Environmental Medicine and Engineering (Ministry of Education), School of Public Health, Southeast University, Nanjing 210096, China; 230198875@seu.edu.cn

**Keywords:** 8-iso-prostaglandin F_2α_ (8-iso-PGF_2α_), oxidative stress, electrospun nanofibers, solid phase extraction, HPLC-MS/MS, urine

## Abstract

The 8-iso-prostaglandin F_2α_ (8-iso-PGF_2α_) biomarker is used as the gold standard for tracing lipid oxidative stress in vivo. The analysis of urinary 8-iso-PGF_2α_ is challenging when dealing with trace amounts of 8-iso-PGF_2α_ and the complexity of urine matrixes. A packed-fiber solid-phase extraction (PFSPE)–coupled with HPLC-MS/MS–method, based on polystyrene (PS)-electrospun nanofibers, was developed for the specific determination of 8-iso-PGF_2α_ in urine and compared with other newly developed LC-MS/MS methods. The method, which simultaneously processed 12 samples within 5 min on a self-made semi-automatic array solid-phase extraction processor, was the first to introduce PS-electrospun nanofibers as an adsorbent for the extraction of 8-iso-PGF_2α_ and was successfully applied to real urine samples. After optimizing the PFSPE conditions, good linearity in the range of 0.05–5 ng/mL with R^2^ > 0.9996 and a satisfactory limit of detection of 0.015 ng/mL were obtained, with good intraday and interday precision (RSD < 10%) and recoveries of 95.3–103.8%. This feasible method is expected to be used for the batch quantitative analysis of urinary 8-iso-PGF_2α_.

## 1. Introduction

At present, the best method to measure oxidative stress in vivo is to detect changes in the levels of the oxidative products of endogenous molecules, such as DNA, protein, or lipids [1]. Isoprostanes are a family of isomers formed by arachidonic acid through enzymatic lipid peroxidation, mediated by prostaglandin intraperoxide synthase (PGHS) [2,3] or chemical lipid peroxidation [4,5,6]. Among these, 8-iso-prostaglandin F_2α_ (8-iso-PGF_2α_) is the most widely recognized lipid peroxidation biomarker [7,8,9] due to its chemical stability [10,11,12,13] and biological activity [14]. 8-iso-PGF_2α_ is excreted in urine [11] and insensitive to dietary-lipid intake [15]. 8-iso-PGF_2α_ has been found to be elevated in many diseases, such as acute lung injury [16], asthma [17], and Alzheimer’s disease [18].

The determination of 8-iso-PGF_2α_ employs enzyme-linked immunosorbent assay (ELISA) [19], gas chromatography-mass spectrometry (GC/MS) [20] and liquid chromatography-mass spectrometry (LC/MS) [21]. The ELISA method is simple, fast, and easy to operate. However, the measured results are often higher than the true value, which may be caused by the cross-reaction between polyclonal antibody and other isoprostaglandin metabolites [12]. GC/MS was considered the gold standard for the determination of 8-iso-PGF_2α_ [22]. However, GC/MS requires time-consuming and laborious prederivational treatment of the sample, which often produces artifacts and contamination [23,24]. Therefore, LC-MS has drawn more attention over the last two decades for its improved specificity and sensitivity, and much easier pretreatment [21,25].

Urine is one of the main excretory systems of the human body and contains a variety of metabolites that are well described under both normal and pathological conditions [26]. Urine collection is a better option than other body fluids because it is more productive and collected in a noninvasive manner. Sample pretreatment is the critical step of 8-iso-PGF_2α_ determination in a complex urine matrix. Packed-fiber solid-phase extraction (PFSPE) is a one-step urine sample pretreatment that integrates extraction, purification, enrichment, and elution and eliminates the steps of nitrogen evaporation and redissolution. In this study, a HPLC-MS/MS method for the specific determination of 8-iso-PGF_2α_ in urine following PFSPE was established. The method introduced polystyrene (PS)-electrospun nanofibers as the adsorbent for 8-iso-PGF_2α_ and processed 12 samples simultaneously within 5 min on a semi-automatic array solid-phase extraction (SPE) processor and was successfully applied to real urine samples. The method was also compared with other newly developed LC/MS/MS methods.

## 2. Results and Discussion

### 2.1. Morphological Characterization of Nanofibers

**Figure 1:**
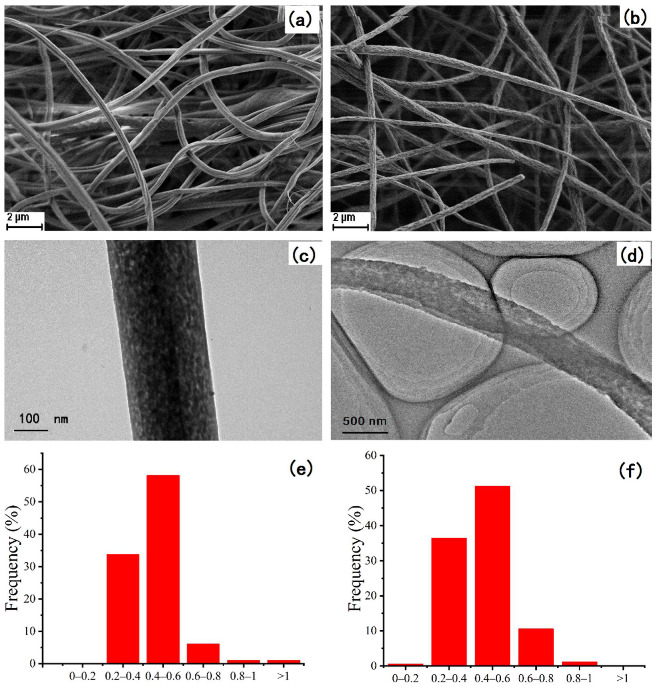
SEM images, TEM images, and the diameter distributions of the nanofibers: (**a**,**c**,**e**) polystyrene (PS) nanofibers and (**b**,**d**,**f**) polystyrene/polypyrrole (PS/PPY) nanofibers.

Scanning electron microscopy (SEM) and transmission electron microscopy (TEM) were carried out on a Hitachi S-3400 N scanning electron microscope (Tokyo, Japan) at an accelerating voltage of 1.0 kV and a FEI, Tecnai G20 transmission electron microscopy (Hillsboro, USA) system at an accelerating voltage of 200 kV. The textural properties of the nanofibers were also studied by a Micromeritics ASAP2020 analyzer (Atlanta, GE, USA). As illustrated in Figure 1, both the polystyrene (PS) nanofibers and polystyrene/polypyrrole (PS/PPY) nanofibers were nanoscale in size so that they could provide large specific surface areas as interaction sites. As illustrated in Table 1, the PS nanofibers had slightly better textural characteristic values of Brunauer-Emmettt-Teller (BET) surface area, pore volume, and pore size than those of the PS/PPY nanofibers.

### 2.2. Optimization of PFSPE Conditions

#### 2.2.1. Comparison between PS and PS/PPY Nanofibers

**Figure 2:**
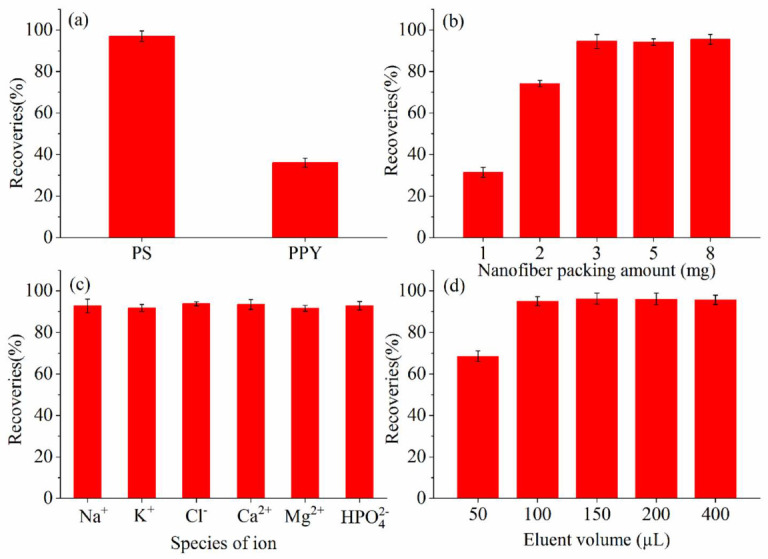
PFSPE condition optimization: (**a**) species of nanofiber; (**b**) ion species; (**c**) species of ion; and (**d**) volume of eluent; error bar = SD (*n* = 3).

The PS and PS/PPY nanofibers were used to extract 8-iso-PGF_2α_ over three replications. As shown in Figure 2a, the extraction recovery of the PS nanofibers was much higher than that of the PS/PPY nanofibers. This indicated that 8-iso-PGF_2α_ was adsorbed on the PS nanofibers mainly through hydrophobic interactions. Due to the polarity of the PPY covering the surface of the PS, the PS/PPY nanofibers are usually used to extract polar compounds [27,28]. Although there are polar groups on the 8-iso-PGF_2α_ molecule, it contains 20 carbon atoms and is less polar and relatively hydrophobic. Representative chromatograms are shown in the Supplementary Materials in Figure S1.

#### 2.2.2. Nanofibers Packing Amount

Different amounts (1, 2, 3, 5 and 8 mg) of PS nanofibers were packed for extracting 8-iso-PGF_2α_ over three replications. As shown in Figure 2b, the extraction recovery increased with the increase in the amount of nanofiber-packing until reaching 3 mg. When the packing amount was greater than 3 mg, the extraction recovery reached equilibrium. An amount of 3 mg of PS nanofibers was enough to extract lipid peroxidation biomarker 8-iso-PGF_2α_ from the urine samples. On the one hand, the nano-size of the electrospun nanofibers endows them with a huge specific surface area. As a result, 8-iso-PGF_2α_ has a large interaction area with the stationary phase and a high distribution coefficient in the solid-liquid phase. Several milligrams of nanofibers are sufficient to complete the adsorption of 8-iso-PGF_2α_. Additionally, an increase in the nanofiber packing amount may lead to an increase in the column pressure and an increase in the amount of solvent required for elution. Therefore, the amount of PS-nanofiber packing was selected as 3 mg.

#### 2.2.3. Species of Ion

Urine contains a large number of electrolytes which are affected by diet as well as physiological and pathological conditions. The influence of major ion species on the extraction efficiency was studied by adding appropriate amounts of Na^+^, K^+^, Cl^−^, Ca^2+^, Mg^2+^, and HPO_4_^2−^ in the loading urine sample with a final concentration of 1 mg/mL for the three replications, respectively. As shown in Figure 2c, the influence was slight and could be ignored.

#### 2.2.4. Volume of Eluent

In order to ensure the effective desorption of 8-iso-PGF_2α_ from the PS nanofibers column, different volumes of methanol (50 µL, 100 µL, 150 µL, 200 µL, and 400 µL) as eluents were tested over the three replications. As shown in Figure 2d, 100 µL methanol was sufficient. Although the extraction recovery slightly improved when the volume of methanol was greater than 100 µL, the increase of eluent volume would reduce the concentration of the target analyte in the eluent, resulting in a decrease in the detection sensitivity. Since the stationary phase column bed was small, the adsorbed 8-iso-PGF_2α_ can be eluted with eluent in microliters. The optimal eluent volume was 100 µL.

### 2.3. Method Validation

#### 2.3.1. Linearity and Sensitivity

Six solutions of 8-iso-PGF_2α_ diluted in artificial urine at concentrations in the range of 0.05–5 ng/mL (0.05, 0.1, 0.2, 0.5, 1, and 5 ng/mL) were analyzed to obtain a calibration curve by comparing the peak area ratio of 8-iso-PGF_2α_ to 8-iso-PGF_2α_-d_4_ against the concentration of 8-iso-PGF_2α_ for five replications. The calibration curve showed good linearity (R^2^ = 0.9996). Limit of detection (LOD) and limit of quantification (LOQ), defined as signal-to-noise ratios of 3:1 and 10:1, were 0.015 ng/mL and 0.05 ng/mL (Table 2) over the five replications, respectively.

#### 2.3.2. Precision and Recovery

The intraday and interday precision were calculated by determining the spiked artificial urine samples at low, medium, and high concentrations of 0.05, 0.5, and 5 ng/mL, following the PFSPE flow described above in quintuplicate for five sequential days. The recovery was estimated as ratios of the measured concentrations (calculated from the standard curve equation) against the spiked concentrations (Table 2). The intraday RSD was 2.1–8.4%. The interday RSD was 4.7–9.2%. The recoveries were 95.3–103.8%.

#### 2.3.3. Matrix Effect

Urine samples from the six healthy volunteers were mixed as blank urine. The stock solution was diluted with the blank urine and water, respectively. All solutions were spiked with internal standard, then treated and analyzed in accordance with the above procedure. The matrix effect (ME) was calculated by comparing the slope of the calibration curve obtained with blank urine as the matrix (Slope_1_) to the slope of the calibration curve obtained with water as the matrix. Matrix effect (Slope_2_) was calculated as follows:

(1) $$\mathrm{ME}=\frac{{\mathrm{Slope}}_{1}}{{\mathrm{Slope}}_{2}}\times 100\%$$

IS normalized matrix factor (Normalized ME), that is, the ratio of the slopes of the two curves corrected by the internal standard. The ME and normalized ME values were 95.4% and 104.2%, respectively. The data were in the 85–115% range, indicating that the influence of the urine matrix was well-controlled [25].

### 2.4. Comparison with Other Methods

Im et al. combined SPE, LLE, and derivatization for urine pretreatment, followed by UHPLC-MS/MS analysis [29]. Commercial Oasis HLB cartridges (3 mL, 60 mg) were used in the SPE procedure. HLB is a macroporous copolymer composed of lipophilic divinyl benzene and hydrophilic N-vinyl pyrrolidone. In order to compare the differences between granular and fibrous adsorbents, the method was also applied to the commercial HLB cartridges (3 mL, 60 mg). The standard solution of 8-iso-PGF_2α_ at a high concentration of 5 ng/mL was processed according to the procedure above. However, the signal obtained in the HPLC-MS/MS instrument was lower than the LOQ. This may be due to the following reasons: firstly, the content of 8-iso-PGF_2α_ was low in the urine sample. Secondly, 100 µL methanol was not enough to elute all of the 8-iso-PGF_2α_. The polymer packed in the HLB column was 60 mg, and typically 3 mL eluent is required to desorb the target compound. Then, steps of nitrogen evaporation and redissolution need to be conducted to achieve enrichment. Therefore, 3 mL of methanol was used instead to elute the HLB cartridges. The eluent was evaporated at 37 °C under a mild stream of nitrogen and then redissolved with 100 µL methanol. The comparative details of the PS nanofibers and HLB as adsorbents are listed in Table 3. Representative chromatograms of 1 mL 5 ng/mL standard solution, processed with PS nanofibers cartridge and HLB cartridge, are shown in Figure S2. Although the HLB polymer particles have a larger specific area and a larger pore volume, their extraction effect was not as good as the PS nanofibers. It was verified that nanofiber-shaped adsorbent performance is better than the particle-shaped adsorbent for the adsorption–desorption of 8-iso-PGF_2α_.

The proposed method was also compared with other newly developed LC-MS/MS methods for 8-iso-PGF_2α_ quantification in Table 4. It was clear that the PFSPE coupled with HPLC-MS/MS established in this work was sensitive enough and more convenient for the determination of urinary 8-iso-PGF_2α_. The method consumed less organic solvent, time, and cost. Unlike other SPE methods that require nitrogen evaporation and redissolution to achieve the effect of enrichment, this method realized the integration of extraction, purification, and enrichment and elution, which greatly simplified the procedure of sample pretreatment and saved a lot of time. PS-electrospun nanofibers were originally introduced as the adsorbent material. Tomov et al. proposed a liquid–liquid extraction (LLE) method to prepare plasma samples [25]; Im et al. combined SPE, LLE, and derivatization for urine pretreatment [29], and Moral et al. [30] proposed an SPE-after-derivatization method. But all these methods consumed a larger volume of organic solvent and required time-consuming nitrogen evaporation and redissolution steps to concentrate the sample. Biagini et al. developed a packed sorbent micro-extraction coupled with UHPLC-MS method for dried-blood spots, which also did not require evaporation and redissolution steps for sample pretreatment and used a small amount of organic solvent, with tests completed in about 10 min. However, it took more than 5 h for the two-fold drying of the dried-blood spot samples for preparation. Only 20 samples could be analyzed per day [21]. However, limitations of our method should also be noted. The filling of nanofibers into the extraction column still needs to be done manually, which still requires standardization and commercial development.

### 2.5. Application to Real Samples

**Figure 3:**
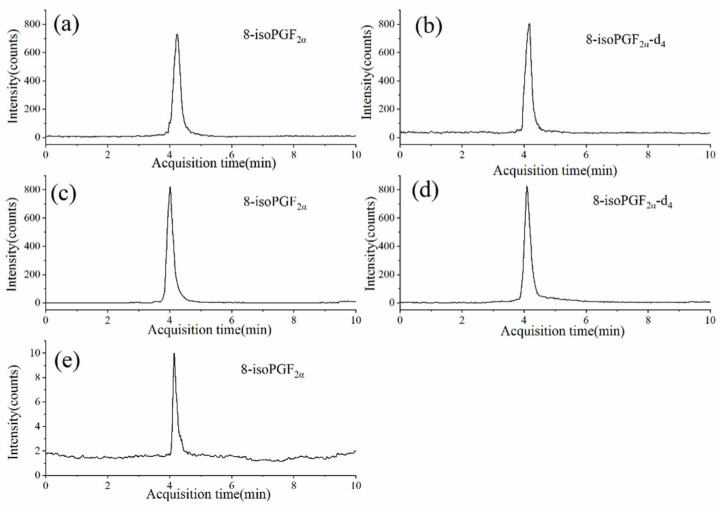
Chromatograms of (**a**) 8-iso-PGF_2α_, (**b**) 8-iso-PGF_2α_-d_4_ in real urine sample after PFSPE; (**c**) 8-iso-PGF_2α_ (0.2 ng/mL), (**d**) 8-iso-PGF_2α_-d_4_ (0.2 ng/mL) in a standard solution with PFSPE; and (**e**) urine sample without PFSPE.

As ASD is 4.2 times more prevalent among boys then girls [31], the genders of the participants were not evenly divided. The urine samples were processed and measured by the method we developed. As shown in Figure 3e, a very small target response was obtained in the real urine sample when only filtered through a 0.22 µm membrane without PFSPE treatment. This might be caused by matrix interference and the trace amount of urinary 8-iso-PGF_2α_. After the PFSPE pretreatment, the response of 8-iso-PGF_2α_ was greatly enhanced (Figure 3a). The creatinine level was measured by a reversed-phase high performance liquid chromatography (RP-HPLC) method according to the Chinese National Standard (WS/T 98-1996) [32]. The urinary 8-iso-PGF_2α_ concentrations of the ASD children and the healthy controls were normalized to the creatinine concentrations as 0.29 ± 0.09 ng/mg creatinine and 0.13 ± 0.03 ng/mg creatinine, respectively. Detailed creatinine normalized 8-iso-PGF_2α_ concentrations of the ASD children and the healthy controls are listed in the Supplementary Materials in Table S1. The results showed that the ASD children had a higher degree of lipid oxidative damage than normal children, which was consistent with the literature [33]. Representative chromatograms of urine samples of the ASD children and healthy volunteers are shown in Supplementary Materials in Figure S3.

## 3. Materials and Methods

### 3.1. Chemicals and Reagents

8-iso-PGF_2α_ and internal standard 8-iso-PGF_2α_-d_4_ were purchased from Cayman Chemical (Ann Arbor, MI, USA), and the chemical structures are shown in Figure S4. HPLC-grade acetonitrile and methanol were purchased from Tedia (Fairfield, OH, USA). The artificial urine (AU) was purchased from Solarbio Science & Technology (Beijing, China). Ultrapure water was used throughout. All the other reagents used were of analytical grade. Dimethylformamide (DMF), tetrahydrofuran (THF), and all of the salts were purchased from Sinopharm Chemical Reagent (Shanghai, China). Polystyrene (PS, Mw = 185,000) were purchased from Shanghai chemical agents Institute (Shanghai, China). Iron (III) chloride (98%) and pyrrole (PY, 98%) were purchased from Alfa Aesar (Haverhill，MA，USA).

Stock solutions of 8-iso-PGF_2α_ (1000 ng/mL) and internal standard 8-iso-PGF_2α_-d_4_ (1000 ng/mL) were prepared in methanol. The working solutions with 8-iso-PGF_2α_ concentrations ranging from 0.05 to 5 ng/mL were prepared by serial dilution of stock solution with water. The working solution of 8-iso-PGF_2α_-d_4_ at 10 ng/mL was diluted weekly with water. All solutions were stored at −20 °C until needed.

### 3.2. Fabrication of PS and PS/PPY Nanofibers

PS nanofibers were electrospun by a modified scheme published by Kang et al. [34]. An amount of 10 mL of 10% (*w/v*) PS solution in DMF and THF (4:6, *v/v*) were loaded into a glass syringe with a 0.4 mm flat tip steel needle. A high-voltage generator was connected to the needle through a copper pin. A piece of tin foil served as the collection screen. The conditions of electrospinning were as follows: high voltage: 22.0 KV; distance between the tip and the collector: 20 cm; solution feeding speed: 1.0 mL/h; temperature: 25 °C; relative humidity: 40%.

Polystyrene/polypyrrole (PS/PPY) nanofibers were electrospun by a modified scheme published by Tian et al. [35]. Brief details are as follows: a PS-nanofiber mat was immersed and rinsed in a 50% ethanol solution. Then, it was soaked in a 0.04 mol/L pyrrole solution, with 0.1 mol/L FeCl_3_ solution added and sonicated at 30 °C overnight for oxidation. PPY was successfully coated on PS nanofibers by in situ polymerization. Finally, the PS/PPY nanofibers were washed with excessive ethanol and ultrapure water for three repetitions and dried in a vacuum oven at 40 °C for 24 h.

### 3.3. Sample Collection

The first morning urine samples of 6 children (5 males and 1 female) with autism spectrum disorder (ASD) and 6 healthy volunteers (5 males and 1 female) aged 4–13 years old, from Nanjing, China, were collected into aseptic urine cups, and then transferred into polypropylene tubes immediately. The samples were stored at −80 °C until analysis. The ASD children were diagnosed by at least two pediatric psychiatry doctors using the Diagnostic and Statistical Manual of Mental Disorders, 5th Edition. The whole study was carried out according to the principles of the Declaration of Helsinki (World Medical Association 2008). Written informed consent was obtained from the guardians of every volunteer. This study was approved by the Ethics Committee of Zhongda Hospital Affiliated to Southeast University.

### 3.4. Sample Pretreatment and PFSPE Procedures

**Figure 4:**
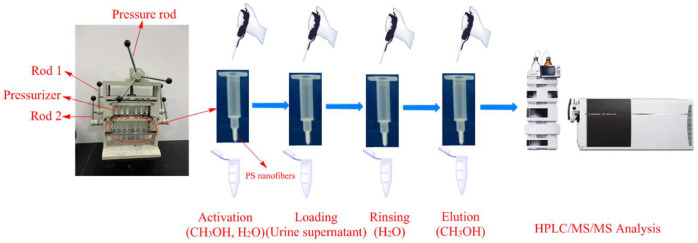
Schematic flow chart of PFSPE procedure.

The SPE column was prepared by packing and compacting 3.0 mg of PS-electrospun nanofibers into the column tip (1.5 mm diameter) with a fine steel rod (0.5 mm diameter). The column was preconditioned with 100 µL of methanol and 100 µL of water, respectively. Freshly thawed urine was centrifuged at 12,000 r/min for 3 min. An aliquot of 1000 μL supernatant was mixed with 20 μL of working 8-iso-PGF_2α_-d_4_ solution by a vortex mixer for 30 s. Then, the mixture was loaded into the SPE column. As shown in Figure 4, a self-made semi-automatic array SPE processor, which can simultaneously pretreat 12 samples with 12 SPE columns, was used. An enlarged view of the device can be found in Supplementary Figure S1 of Zhao’s article [28]. The pressurizers converted from syringes were installed in the upper plate and the SPE columns were installed in the lower plate. The lower plate can be moved and fixed by operating Rod 1 and Rod 2 at the same time. Air pressure was provided by rotating the pressure rod to drive the push rod of the pressurizer, controlling the solution to pass through the column at a rate of 5 s per drop. The column was rinsed with 100 µL of water for 3 repetitions. Finally, the column was eluted with 100 µL of methanol, and 20 µL of the eluent was injected for detection immediately.

### 3.5. HPLC-MS/MS Analysis

An Agilent 1260 Infinity LC system equipped with a 6460 Triple Quad mass spectrometer (Agilent Technologies, Palo Alto, Santa Clara, CA, USA) was used for the analysis. Electrospray ion source: ESI; Ion polarity: negative ion; Monitoring mode: multiple response monitoring (MRM); Chromatographic column: Agilent Eclipse xdb-C_18_ (3.5 μm, 4.6 mm × 150 mm); Mobile phase: acetonitrile: 0.1% formic acid aqueous solution (90:10), Flow rate: 0.4 mL/min; Column temperature: 30 °C; Injection volume: 10 μL. Gas temperature: 300 °C; Gas flow: 11 L/min, Nebulizer gas pressure: 15 psi; Capillary voltage: −4000 V; Fragmentor voltage: 152 V. The quantitative ion, collision energy, and retention times are listed in Table 5.

## 4. Conclusions

A packed, PS-electrospun-nanofiber SPE coupled with HPLC-MS/MS method for rapid determination of urinary 8-iso-PGF_2α_ was developed in this paper. Under optimized conditions, a one-step pretreatment of the sample, which integrated extraction, purification, enrichment, and elution, was realized, with the steps of nitrogen evaporation and re-dissolution abandoned. The method can simultaneously process 12 samples within 5 min on a self-made semi-automatic array SPE processor and has been successfully applied to real urine samples. Therefore, this feasible method can be expected to be used for batch quantitative analysis of lipid damage biomarker 8-iso-PGF_2α_ in urine.

## Figures and Tables

**Table 1:** Textural properties of nanofibers.

Nanofibers	BET Surface Area (m^2^/g)	Pore Volume (cm^3^/g)	Pore Size (nm)
PS	18.72	0.18	39.33
PS/PPY	15.40	0.11	28.04

BET: Brunauer-Emmett-Teller; PS: polystyrene; PS/PPY: polystyrene/polypyrrole nanofibers.

**Table 2:** Analytical parameters of the method (*n* = 5).

Analyte	Linear Range (ng/mL)	R^2^	LOD (ng/mL)	LOQ (ng/mL)	Spiked Concentration (ng/mL)	RSD (%)		Recovery (%)
						Intra-Day	Inter-Day	
8-iso-PGF_2α_	0.05–5	0.9996	0.015	0.05	0.05	8.4	9.2	103.8 ± 9.3
					0.5	5.3	6.8	97.5 ± 4.9
					5	2.1	4.7	95.3 ± 4.8

**Table 3:** Comparison between PS nanofibers and HLB as adsorbents.

Adsorbent	Adsorbent Amount (mg)	Size	Specific Surface Area (m^2^/g)	Pore Size (nm)	Pore Volume (cm^3^/g)	Evaporation	Recovery (%)
PS nanofibers	3	500 nm	18.72	39.33	0.18	NO	102.4
HLB *	60	28.3 µm	808	8.3	1.31	YES	78.7

* HLB: Macroporous polymer composed of lipophilic divinyl benzene and hydrophilic N-vinyl pyrrolidone.

**Table 4:** Comparison of LC-MS/MS methods for 8-isoPGF2α analysis.

Method	Sample	Sample Amount (µL)	Sample Preparation	Pretreatment Time	Organic Solvent (mL)	LOD (ng/mL)	Recovery (%)	Evaporation	Ref.
HPLC-MS/MS	Plasma, urine	300	Hydrolysis, immunoaffinity column	>60 min	>2	0.0005	78–102, 75–99	YES	[24]
UHPLC-MS/MS	Dried blood spots	50	Packed sorbent silica-C_18_ Barrel Insert and Needles	>5 h for spot dry, 10 min for SPE	0.58	0.015	89.1–109.5	YES	[21]
HPLC-MS/MS	Plasma	500	LLE	>1 h	>4	-	59.2–68.5	YES	[25]
UHPLC-MS/MS	Urine	100	SPE (OASIS HLB *), Incubation, derivatization, LLE	>3.5 h	>8	-	101.4	YES	[29]
HPLC-MS/MS	Urine	500	Incubation, derivatization, SPE (ABS ElutNexus **)	>30 min	>4	0.013	-	YES	[30]
HPLC-MS/MS	Urine	1000	PFSPE (PS nanofibers)	≈5 min	0.2	0.015	92.3–104.9	NO	this study

* OASIS HLB cartridges: Macroporous copolymer cartridges formed by the polymerization of lipophilic divinyl benzene and hydrophilic N-vinyl pyrrolidone. ** ABS ElutNexus cartridges: Polymer extraction cartridges formed by polystyrene divinylbenzene and polymethyl methacrylate.

**Table 5:** Quantitative ion, collision energy, and retention times of 8-iso-PGF_2α_ and 8-iso-PGF_2α_-d_4_.

Compound	Quantitative Ion (*m/z*)	CE (V)	Retention Time (min)
8-iso-PGF_2α_	353.2→309.1	14	4.22
	353.2→193	22	
8-iso-PGF_2α_-d_4_	357.2→197.2	22	4.16

## Data Availability

The data presented in this study are available on reasonable request from the corresponding author.

## Supplementary Materials

The following supporting information can be downloaded at: https://www.mdpi.com/article/10.3390/molecules27144417/s1, Figure S1: Representative chromatograms of PFSPE condition optimization. 1 mL 1 ng/mL standard solution processed with (a) PS nanofibers cartridge and (b) PS/PPY cartridge. Figure S2: Representative chromatograms of 1 mL 5 ng/mL standard solution processed with (a) PS nanofibers cartridge and (b) HLB cartridge; Figure S3: Representative chromatograms of urine of (a) ASD children and (b) healthy control; Figure S4: Structures of 8-iso-PGF2a and 8-iso-PGF2a-d4; Table S1: The creatinine normalized 8-iso-PGF2α concentrations of ASD children and healthy controls.

## Sample Availability

Samples of the compounds are not available from the authors.

## References

[1] Kadiiska M.B., Gladen B.C., Baird D.D., Germolec D., Graham L.B., Parker C.E., Nyska A., Wachsman J.T., Ames B.N., Basu S. (2005). Biomarkers of oxidative stress study II: Are oxidation products of lipids, proteins, and DNA markers of CCl4 poisoning?. Free Radic. Biol. Med..

[2] Van ’t Erve T.J., Lih F.B., Kadiiska M.B., Deterding L.J., Eling T.E., Mason R.P. (2015). Reinterpreting the best biomarker of oxidative stress: The 8-iso-PGF(2alpha)/PGF(2alpha) ratio distinguishes chemical from enzymatic lipid peroxidation. Free Radic. Biol. Med..

[3] Leitinger N., Blazek I., Sinzinger H. (1997). The generation and actions of isoprostanes. Thromb. Res..

[4] Morrow J.D., Harris T., Roberts L.J. (1990). Noncyclooxygenase oxidative formation of a series of novel prostaglandins—Analytical ramifications for measurement of eicosanoids. Anal. Biochem..

[5] Yin H., Xu L., Porter N.A. (2011). Free radical lipid peroxidation: Mechanisms and analysis. Chem. Rev..

[6] Yin H., Porter N.A., Morrow J.D. (2005). Separation and identification of F2-isoprostane regioisomers and diastereomers by novel liquid chromatographic/mass spectrometric methods. J. Chromatogr. B Anal. Technol. Biomed. Life Sci..

[7] Montuschi P., Barnes P.J., Roberts L.J. (2004). Isoprostanes: Markers and mediators of oxidative stress. FASEB J..

[8] Roberts L.J., Milne G.L. (2009). Isoprostanes. J. Lipid Res..

[9] Dalle-Donne I., Rossi R., Colombo R., Giustarini D., Milzani A. (2006). Biomarkers of oxidative damage in human disease. Clin. Chem..

[10] Basu S. (2008). F2-isoprostanes in human health and diseases: From molecular mechanisms to clinical implications. Antioxid. Redox Signal..

[11] Roberts L., Morrow J. (2000). Measurement of F2-isoprostanes as an index of oxidative stress in vivo. Free Radic. Biol. Med..

[12] Milne G.L., Dai Q., Roberts L.J. (2015). The isoprostanes—25 years later. Biochim. Biophys. Acta.

[13] Morrow J.D., Hill K., Burk R., Nammour T., Badr K., Roberts L. (1990). A series of prostaglandin F2-like compounds are produced in vivo in humans by a non-cyclooxygenase, free radical-catalyzed mechanism. Proc. Natl. Acad. Sci. USA.

[14] Basu S. (2010). Bioactive eicosanoids: Role of prostaglandin F(2alpha) and F(2)-isoprostanes in inflammation and oxidative stress related pathology. Mol. Cells.

[15] Gopaul N.K., Halliwell B., Anggard E.E. (2000). Measurement of plasma F2-isoprostanes as an index of lipid peroxidation does not appear to be confounded by diet. Free Radic. Res..

[16] Janssen L. (2001). Isoprostanes:an overview and putative roles in pulmonary pathophysiology. Am. J. Physiol..

[17] Duchene B., Caffry S., Kaminsky D.A., Que L.G., Poynter M.E., Dixon A.E. (2021). Functional significance of 8-isoprostanes in sinonasal disease and asthma. Respir. Med..

[18] Ferre-Gonzalez L., Pena-Bautista C., Baquero M., Chafer-Pericas C. (2022). Assessment of Lipid Peroxidation in Alzheimer’s Disease Differential Diagnosis and Prognosis. Antioxidants.

[19] Proudfoot J., Barden A., Mori T., Burke V., Croft K., Beilin L., Puddey I. (1999). Measurement of urinary F-2,-isoprostanes as markers of in vivo lipid peroxidation—A comparison of enzyme immunoassay with gas chromatography_mass spectrometry. Anal. Biochem..

[20] Wiswedel I. (2009). F(2)-isoprostanes: Sensitive biomarkers of oxidative stress in vitro and in vivo: A gas chromatography-mass spectrometric approach. Methods Mol. Biol..

[21] Biagini D., Antoni S., Lomonaco T., Ghimenti S., Salvo P., Bellagambi F.G., Scaramuzzo R.T., Ciantelli M., Cuttano A., Fuoco R. (2020). Micro-extraction by packed sorbent combined with UHPLC-ESI-MS/MS for the determination of prostanoids and isoprostanoids in dried blood spots. Talanta.

[22] Smith K.A., Shepherd J., Wakil A., Kilpatrick E.S. (2011). A comparison of methods for the measurement of 8-isoPGF(2alpha): A marker of oxidative stress. Ann. Clin. Biochem..

[23] Morrow J.D., Frei B., Longmire A.W., Gaziano J.M., Lynch S.M., Shyr Y., Strauss W.E., Oates J.A., Roberts L.J. (1995). Increase in circulating products of lipid-peroxidation (F-2-Isoprostanes) in smokers—smoking as a cause of oxidative damage. N. Engl. J. Med..

[24] Sircar D., Subbaiah P.V. (2007). Isoprostane measurement in plasma and urine by liquid chromatography-mass spectrometry with one-step sample preparation. Clin. Chem..

[25] Tomov D.G., Bocheva G., Divarova V., Kasabova L., Svinarov D. (2021). Phase separation liquid-liquid extraction for the quantification of 8-iso-Prostaglandin F2 Alpha in human plasma by LC-MS/MS. J. Med. Biochem..

[26] Decramer S., de Peredo A.G., Breuil B., Mischak H., Monsarrat B., Bascands J.-L., Schanstra J.P. (2008). Urine in Clinical Proteomics. Mol. Cell. Proteom..

[27] Sun Y., Xie L., Feng F., Han Q., Wei L., Tang Z., Kang X. (2020). Simultaneous analysis of two urinary biomarkers of oxidative damage to DNA and RNA based on packed-fiber solid phase extraction coupled with high-performance liquid chromatography. J. Chromatogr. B Anal. Technol. Biomed. Life Sci..

[28] Zhao R., Chu L., Wang Y., Song Y., Liu P., Li C., Huang J., Kang X. (2017). Application of packed-fiber solid-phase extraction coupled with GC–MS for the determination of short-chain fatty acids in children’s urine. Clin. Chim. Acta.

[29] Im E., Lew B.L., Lee M.Y., Lee J., Paeng K.J., Chung B.C. (2019). Simultaneous determination of androgens and prostaglandins in human urine using ultra-high-performance liquid chromatography-tandem mass spectrometry. J. Chromatogr. B Anal. Technol. Biomed. Life Sci..

[30] Martinez-Moral M.P., Kannan K. (2022). Analysis of 19 urinary biomarkers of oxidative stress, nitrative stress, metabolic disorders, and inflammation using liquid chromatography-tandem mass spectrometry. Anal. Bioanal. Chem..

[31] Maenne M.J., Shaw K.A., Bakian A.V., Bilder D.A., Durkin M.S., Esler A. (2021). Prevalence and Characteristics of Autism Spectrum Disorder Among Children Aged 8 Years-Autism and Developmental Disabilities Monitoring Network, 11 Sites, United States, 2018. Surveill. Summ..

[32] China Mohotpsro (1997). Urine—Determination of creatinine—Reversed-phase High Performance Liquid Chromatographic Method.

[33] Bjorklund G., Meguid N.A., El-Bana M.A., Tinkov A.A., Saad K., Dadar M., Hemimi M., Skalny A.V., Hosnedlova B., Kizek R. (2020). Oxidative Stress in Autism Spectrum Disorder. Mol. Neurobiol..

[34] Kang X., Pan C., Xu Q., Yao Y., Wang Y., Qi D., Gu Z. (2007). The investigation of electrospun polymer nanofibers as a solid-phase extraction sorbent for the determination of trazodone in human plasma. Anal. Chim. Acta.

[35] Tian T., Deng J., Xie Z., Zhao Y., Feng Z., Kang X., Gu Z. (2012). Polypyrrole hollow fiber for solid phase extraction. Analyst.

