# Supplementary material for: Packed-Fiber Solid Phase-Extraction Coupled with HPLC-MS/MS for Rapid Determination of Lipid Oxidative Damage Biomarker 8-Iso-Prostaglandin F2α in Urine
Source: Molecules. 2022 Jul 10;27(14):4417. doi: 10.3390/molecules27144417 (PMC9318247; doi:10.3390/molecules27144417)
Supplement: Supplementary file 1 [file molecules-27-04417-s001.zip › molecules-1798755-supplementary.pdf]

Article

Packed-fiber Solid Phase Extraction Coupled with HPLC-MS/MS for Rapid Determination of Lipid Oxidative Damage Biomarker 8-iso-prostaglandin $F_{2\alpha}$ in Urine

Ying Sun [1], Yan Yan [2], Xuejun Kang [1, 2*]

[1] Key Laboratory of Child Development and Learning Science, Ministry of Education, School of Biological Science & Medical Engineering, Southeast University, Nanjing, 210096, China; 230189182@seu.edu.cn;

[2] Key Laboratory of Environmental Medicine and Engineering (Ministry of Education), School of Public Health, Southeast University, Nanjing 210096, China; 230198875@seu.edu.cn

* Correspondence: 101006214@seu.edu.cn; Tel.: +86-025-83795664-1011.

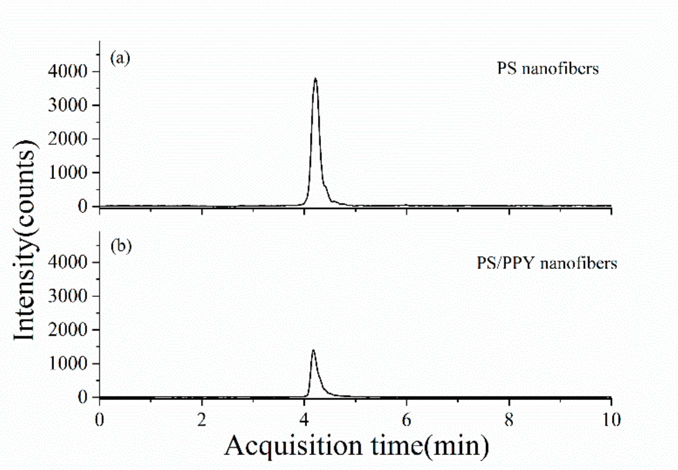

Figure S1. Representative chromatograms of PFSPE condition optimization.1 mL 1 ng/mL standard solution processed with (a) PS nanofibers cartridge and (b) PS/PPY cartridge.

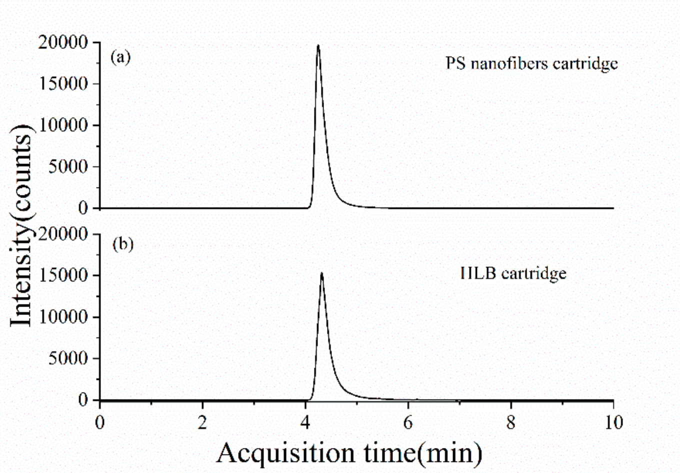

Figure S2. Representative chromatograms of 1 mL 5 ng/mL standard solution processed with (a) PS nanofibers cartridge and (b) HLB cartridge.

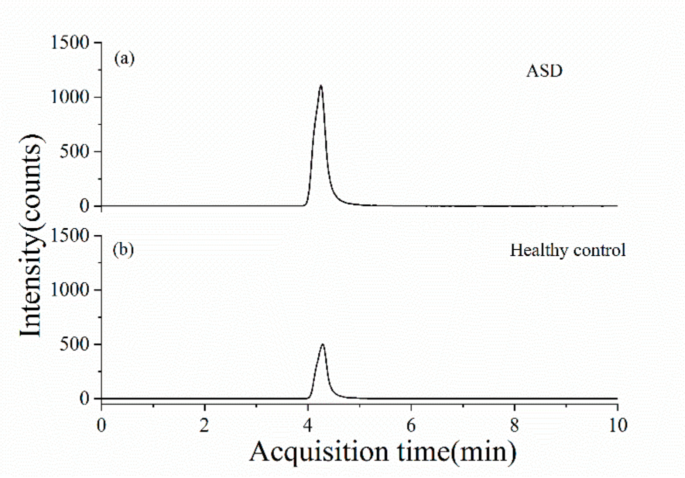

Figure S3. Representative chromatograms of urine of (a) ASD children and (b) healthy control.

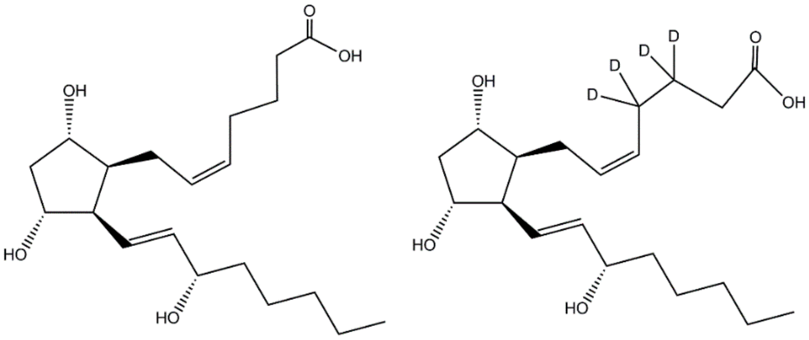

8-iso-PGF2α 8-iso-PGF2α–d4

Figure S4. Structures of 8-iso-PGF$_{2a}$ and 8-iso-PGF$_{2a}$-d$_4$.

Table S1. The creatinine normalized 8-iso-PGF$_{2\alpha}$ concentrations of ASD children and healthy controls.

Group	Concentration (ng/mg Cr)					
	1	2	3	4	5	6
ASD	0.17	0.25	0.34	0.37	0.40	0.21
Control	0.11	0.09	0.13	0.11	0.16	0.17
